# Microbial communities in aerosol generated from cyanobacterial bloom-affected freshwater bodies: an exploratory study in Nakdong River, South Korea

**DOI:** 10.3389/fmicb.2023.1203317

**Published:** 2023-07-13

**Authors:** Jinnam Kim, GyuDae Lee, Soyeong Han, Min-Ji Kim, Jae-Ho Shin, Seungjun Lee

**Affiliations:** ^1^Major of Food Science & Nutrition, Division of Food Science, College of Fisheries Science, Pukyong National University, Busan, Republic of Korea; ^2^Department of Applied Biosciences, Kyungpook National University, Daegu, Republic of Korea; ^3^NGS Core Facility, Kyungpook National University, Daegu, Republic of Korea

**Keywords:** harmful algal blooms, microcystin, *Microcystis*, aerosol, Nakdong River, metagenomics, microbiome

## Abstract

Toxic blooms of cyanobacteria, which can produce cyanotoxins, are prevalent in freshwater, especially in South Korea. Exposure to cyanotoxins via ingestion, inhalation, and dermal contact may cause severe diseases. Particularly, toxic cyanobacteria and their cyanotoxins can be aerosolized by a bubble-bursting process associated with a wind-driven wave mechanism. A fundamental question remains regarding the aerosolization of toxic cyanobacteria and cyanotoxins emitted from freshwater bodies during bloom seasons. To evaluate the potential health risk of the aerosolization of toxic cyanobacteria and cyanotoxins, the objectives of this study were as follows: 1) to quantify levels of microcystin in the water and air samples, and 2) to monitor microbial communities, including toxic cyanobacteria in the water and air samples. Water samples were collected from five sites in the Nakdong River, South Korea, from August to September 2022. Air samples were collected using an air pump with a mixed cellulose ester membrane filter. Concentrations of total microcystins were measured using enzyme-linked immunosorbent assay. Shotgun metagenomic sequencing was used to investigate microbial communities, including toxic cyanobacteria. Mean concentrations of microcystins were 960 μg/L ranging from 0.73 to 5,337 μg/L in the water samples and 2.48 ng/m^3^ ranging from 0.1 to 6.8 ng/m^3^ in the air samples. In addition, in both the water and air samples, predominant bacteria were *Microcystis* (PCC7914), which has a microcystin-producing gene, and *Cyanobium*. Particularly, abundance of *Microcystis* (PCC7914) comprised more than 1.5% of all bacteria in the air samples. This study demonstrates microbial communities with genes related with microcystin synthesis, antibiotic resistance gene, and virulence factors in aerosols generated from cyanobacterial bloom-affected freshwater body. In summary, aerosolization of toxic cyanobacteria and cyanotoxins is a critical concern as an emerging exposure route for potential risk to environmental and human health.

## 1. Introduction

Events of harmful algal blooms (HABs) caused by cyanobacteria in freshwater (e.g., rivers, lakes, and reservoirs) are a critical issue worldwide (Lu et al., 2020). Climate change may exacerbate the dangers associated with increased occurrence and severity of HABs (Lee et al., 2021). Furthermore, human activities, such as agricultural practice, may create optimal conditions for promoting the growth of toxic cyanobacteria (Hur et al., 2013). Cyanobacteria are a group of oxygenic photosynthetic bacteria widely distributed in freshwater and marine environments (Gonçalves et al., 2016). Cyanobacteria, which do not contain a membrane-bound nucleus, mitochondria, and chloroplasts, are primitive micro-organisms with an intermediate structure between bacteria and plants (Hachicha et al., 2022). Despite formerly being considered algae due to their photosynthetic ability, cyanobacteria are currently classified as bacteria (prokaryotic) (Oren, 2011; Palinska and Surosz, 2014). Several cyanobacteria can produce toxic compounds (known as cyanotoxins) that are problematic when freshwater is used for drinking, agricultural, and recreational purposes (Wood and Dietrich, 2011). Animals and humans can become exposed to cyanotoxins, including anatoxins, cylindrospermopsins, microcystins (MCs), nodularins, and saxitoxins, via inhalation, ingestion, and dermal contact; in particular, MCs are selectively hepatotoxic in fish, birds, and mammals (Dawson, 1998). MC poisoning can cause hepatocyte necrosis and hemorrhage (Bhattacharya et al., 1997). MCs have also been associated with tumor promotion over long-term exposure (Ito et al., 1997). Recent studies focused on human exposure to aerosolized MCs that are significantly related with proinflammatory response in human airway epithelium (Orrell, 2022; Labohá et al., 2023). Both toxic cyanobacteria and MCs either float around the water column or attach to the surface and subsequently enter the atmosphere when they are undulated by the wind or introduced by waves, rainfall, and ship traffic. In a study measuring MC concentrations in the nasal mucosa of residents during a cyanobacterial bloom in a river in Florida, 115 of 121 participants (95.0%) had MC concentrations above the detection limit, with an average concentration of 0.61 + 0.75 μg/L. This result suggests that aerosolization of cyanotoxins is an important pathway for evaluating potential health risk (Schaefer et al., 2020). In addition, in a previous study examining the toxicity of aerosolized MCs to mice, the risk of inhaled MCs was found to be ten times higher than that of orally administered MCs (Benson et al., 2005). Furthermore, in the United Kingdom, respiratory illnesses such as pneumonia have been reported in many people canoeing in reservoirs where *Microcystis* HABs have progressed (Stewart et al., 2006). The results of Sharma et al. (2007) suggest that inhaling cyanotoxins may have worse effects than ingesting cyanotoxins. These studies have emphasized the importance of monitoring cyanotoxins in the air (Olson et al., 2020; Lad et al., 2022; Vigar et al., 2022; Rogers and Stanley, 2023).

The Nakdong River is the longest river in the Republic of Korea (hereafter South Korea), with a total length of 525 km and a watershed area of 23.859 km^2^ (Ryu et al., 2016). Water from the Nakdong River has been used as a source of tap water by two metropolitan cities (Daegu and Busan) and several small cities, for recreational activities, and for agricultural purpose (Kim et al., 2021). However, the Nakdong River is afflicted with eutrophication with nitrogen (N) and phosphorus (P), which are critical factors for triggering occurrence of HABs (Kim et al., 2021). Furthermore, since 2012, eight large artificial weirs have been constructed along a 200 km section of the Nakdong River. These weirs reduce the flow velocity and artificially alter the water flow to form stagnant waterbodies, which can lead to the flourishing of cyanobacteria and occurrence of HABs (Choi et al., 2002; Lee et al., 2018; Park et al., 2021). HABs caused by cyanobacteria have occurred frequently in the Nakdong River (Kim et al., 2019). As the Nakdong River is a major source of drinking water, agricultural water, and commercial water, HABs that can produce cyanotoxins pose a serious problem for water use (Park et al., 2021). In particular, cyanobacteria and their toxins may be aerosolized and exposed to people through recreational activities, ship operation, and wind friction (Tesson et al., 2016). However, research on the aerosolization of toxic cyanobacteria and MCs is insufficient in the context of the Nakdong River in South Korea.

Furthermore, eutrophication and HABs can impact the aquatic environment in several ways. Firstly, eutrophication may promote the survival and proliferation of pathogens in aquatic environments (Smith and David, 2009). Recently, it has been proposed that HABs, triggered by eutrophication, could also contribute to the emergence and dissemination of antibiotic resistance genes in aquatic environments (Zhang et al., 2020; Li et al., 2021). Therefore, it is essential to examine the presence of both the pathobiome and the resistome. The concept of pathobiome refers to the collection of host-associated organisms associated with potential health risk (Bass et al., 2019). The resistome refers to all antibiotic resistance genes (ARGs) and their precursors in both pathogenic and nonpathogenic bacteria. The resistome serves as a comprehensive overview of ARGs, accounting for different resistance mechanisms and their potential evolution within microbial communities (Kim and Cha, 2021). In this study, metagenomic analysis was used to examine profiles of microbial communities in water and aerosol samples during HAB events in Nakdong River, South Korea, with quantification of MCs. In addition, the relationship between environmental factors (wind speed, precipitation, air temperature, and humidity) and aerosolization of toxic cyanobacteria and MCs was examined. We aimed to understand the microbial communities, including cyanobacterial community, ARGs, and virulence factors, in aerosols emitted from the Nakdong River during bloom seasons. This study may also provide evidence of potential health risk related to aerosolization of toxic cyanobacteria and cyanotoxins to which humans can be exposed via inhalation.

## 2. Materials and methods

### 2.1. Sample collection, meteorological data, and DNA extraction

Water and aerosol samples were collected from the five sites of Nakdong River (Daedong Wharf, Samnakdunchi, Hwawon Amusement Park, Leports Valley, Hapcheon Hakri Reservoir) on August 30 and September 2, 2022. A sterile mixed cellulose ester membrane filter (diameter 37 mm, pore size 0.8 μm, Merck Millipore, Billerica, MA, USA) was used for the aerosol sampling. The filter paper was placed in an aluminum filter holder with a stainless-steel support connected with a silicone tube to a vacuum pump (Model SIP-32 L, Sibata Scientific Technology Ltd., Tokyo, Japan). The flow rate of sampling was adjusted to about 0.02 m^3^/min and aerosol samples were collected for a total of 4 h (total volume: 4.8 m^3^). To validate the quality control of aerosol samples, additional aerosol samples were collected in a sterile environment (clean bench) for 8 h. The 16S rRNA and *mcyE* genes were amplified using polymerase chain reaction (PCR) method from extracted DNA of the aerosol samples and the samples of quality control. Gel electrophoresis was performed to confirm microbial contamination.

Water samples (100 mL per hour for 4 h, total volume: 400 mL) were collected from the surface water of the river using a van dorn water sampler (depth: 30 cm, PDNN19080900002, Daihan chemlab, South Korea). For delivering the water and aerosol samples, the samples were kept at 4°C. In addition, meteorological data (i.e., wind speed, precipitation, air temperature and humidity) were obtained by Korea Meteorological Administration (https://www.kma.go.kr/eng/index.jsp).

In the laboratory (Pukyong National University, Busan, South Korea), water samples were filtered with a MultiVac 610-MS-T Multi-Branch Filtration system; each 100 mL water sample was filtered through a sterile 0.22 μm, 47 mm Whatman Nuclepore Hydrophilic Membrane filter (Wilson, 2022). For extracting microbial DNA from the filters (water and aerosol samples), the DNeasy PowerSoil Pro kit (QIAGEN, Germany) was used according to the manufacturer’s instructions.

### 2.2. Measurement of water quality

Water parameters, including conductivity, dissolved oxygen (DO), and pH, were measured using a water quality meter (AZ-8603, AZ Instrument, Taiwan). The biochemical oxygen demand (BOD) value was also examined (Zhao et al., 2019). Concentrations of total nitrogen (N) and total phosphorus (P) were measured using Hach Digital Reactor Block 200 (Hach Co., Loveland, CO, USA) following by Persulfate Digestion Method 10,071, and USEPA PhosVer 3 with Acid Persulfate Digestion Method 8,190, respectively.

MCs were extracted from the air filters and measured according to the previous study (Murby et al., 2016; Swe et al., 2021). Briefly, each sample was conducted with frozen and thawed at three times, and then the samples were applied with sonicating for 1 min and vortexing for 30 s. For measuring levels of MCs in the water samples, US EPA Method 546 was used. Briefly, for cell lysing, the samples were conducted with frozen and thawed at three times. MC concentration was analyzed in triplicate with an enzyme-linked immunosorbent assay (ELISA) kit (PN.520011SAES, Eurofins Abraxis, Warminister, PA, USA) according to the manufacturer’s protocol. The detection limit of the kit is 0.016 μg/L (detection range: 0.05 to 5 μg/L). For measuring high concentrations of MCs (more than 5 μg/L) in the water samples, the samples were diluted with double-distilled water and then levels of MCs were re-measured.

### 2.3. Quantification of MC-producing *Microcystis*

Levels of MC-producing *Microcystis* in water and aerosol samples were quantified with Quantstudio 1 Real-Time PCR system (Applied Biosystems, Foster City, CA, USA) by targeting the *mcyE* gene that encodes *Microcystis*-specific MC production with the set of primers and PCR conditions from a previous study (Sipari et al., 2010). PCR reaction solutions contained TOPreal™ qPCR 2x PreMIX with SYBR green (Enzynomics, Daejeon, South Korea), 10 μM of each primer and the extracted DNA (total volume was 20 μL). The thermal cycling conditions were 50°C for 2 min, 95°C for 10 min, 40 cycles of 95°C for 30 s, and 62°C for 1 min, and by a melting curve stage of 95°C for 15 s and 60°C for 1 min (Lee et al., 2021). The output data were analyzed by associated software (Design and Analysis Software 2.6.0, Applied Biosystems, CA, USA). All experiments were performed in triplicate.

### 2.4. Library preparation and shotgun metagenomic sequencing

For the aerosol filtered samples, amplification was conducted using REPLI-g Single Cell Kit (Qiagen, Germany) according to manufacturer’s protocol to ensure sufficient concentration of DNA. DNA concentration and quality were measured using Qubit™ Flex Fluorometer (Thermo Fisher Scientific, USA) and Nanodrop One spectrophotometer (Thermo Fisher Scientific, USA), respectively. The sequencing library was prepared using the MGIEasy FS DNA Library Prep Kit, Circularization Module, and DNBSEQ-G400RS High-throughput Sequencing Kit PE100 (MGI Tech, China) following the manufacturer’s instructions. Shotgun metagenome sequencing was performed at NGS Core facility (Kyungpook National University, Daegu, Korea). The raw sequences were deposited in the National Center for Biotechnology Information (NCBI) SRA dataset under BioProject accession number PRJNA949880 (https://www.ncbi.nlm.nih.gov/bioproject/PRJNA949880).

### 2.5. Preprocessing of sequencing raw data and taxonomy classification

For trimming low quality sequences of raw reads, Trimmomatic (v.0.39) was performed (Bolger et al., 2014). Reads were scanned with a 50-base wide sliding window, and average quality per base below 28 were trimmed. Using quality filtered reads, taxonomy classification was conducted using Kraken2 (Wood et al., 2019). Custom database was built from NCBI sequences which were include bacterial, fungal, archaeal, and viral genomes. Additional estimation was performed using Bracken to accurately predict the abundance of microbial features at each taxonomic rank based on the resulting output report (Lu et al., 2017).

### 2.6. Shotgun metagenome assembly and annotation of functional genes

Preprocessed reads from all the samples were merged for co-assembly with MEGAHIT (v1.2.9) (Li et al., 2015). The minimum k-mer was set to 27 and did not add mercy k-mers. We utilized the assembled contigs to carry out two processes: taxa identification of each contigs using Kraken2 and gene prediction. Protein-coding sequences (CDS) were predicted using Prodigal (v2.6.3), and redundant protein sequences with more than 95% identity were clustered using CD-HIT. (v4.8.1) (Li and Godzik, 2006). RPKM (reads per kilobase of exon per million reads mapped) of each predicted gene was calculated using BBMap (Bushnell, 2014). In case of ARGs, the abundance was calculated by following equation:


$$\begin{array}{l} ARGsabundance=\\ \overset{n}{\underset{i}{\sum }}\frac{N_{i\left(ARG-Likesequence\right)}\times \frac{L_{reads}}{L_{i\left(ARG\;referencesequence\right)}}}{N_{16S\;sequence}\times \frac{L_{reads}}{L_{16S\;sequence}}} \end{array}$$

$N_{i\left(ARG-Likesequence\right)}$ is the total number of reads which predicted to ARG with CARD reference, $L_{i\left(ARG\;referencesequence\right)}$ is each read length of predicted ARG, $L_{reads}$ is the average read length of preprocessed raw read, $N_{16S\;sequence}$ is the total number of reads which encoding 16S rRNA gene using SILVA database from Kraken2 custom database. $L_{16S\;sequence}$ is the average length of the 16S rRNA gene sequence in the database (1,080 bp). Gene annotation for functional analysis was done using DIAMOND (v2.0.15.153) (Buchfink et al., 2015). For functional analysis, gene annotation of predicted CDS was carried out using DIAMOND (v2.0.15.153) with parameters set to 70% identity and 80% query coverage. The microcystin biosynthetic gene cluster (BGC0001017: *Microcystis aeruginosa* PCC 7806) was retrieved from the Minimum Information about a Biosynthetic Gene Cluster (MIBiG) database, while the CARD (Alcock et al., 2019) and VFDB (Liu et al., 2022) databases were utilized for resistome and pathobiome analysis, respectively.

### 2.7. Statistical analysis

Statistical analyses and visualization were processed in R studio (v3.6.3). Importing of microbiome data including taxonomy information, feature table, and metadata was conducted using phyloseq package. Diversity analysis and visualized using vegan and ggplot package, respectively. Bray–curtis dissimilarity was computed for beta diversity analysis and visualized using principal coordinate analysis (PCoA). To statistically test the sample distance between freshwater and aerosol samples, adonis test in the vegan package for permutational multivariate analysis of variance (PERMANOVA) was conducted. Alpha diversity indices including Chao1 and Simpson were calculated using the microbiome package. To perform statistical tests comparing the indices between freshwater and aerosol groups, the Wilcoxon rank-sum test was conducted. The core microbiome and genes were filtered using microbiome package to elements that were found in more than 50% of each group and enriched by more than 1 and 0.001%, respectively.

## 3. Results

### 3.1. Quantification of total MCs and MC-producing *Microcystis*

Total MCs in the water and aerosol samples were measuring (Table 1). The mean concentration of MCs in freshwater samples was approximately 1,158 μg/L. This value was about 48 times higher than that of the world health organization (WHO) recreational guideline (24 μg/L). The highest concentration of MCs was the FW5 sample (5337.39 μg/L). In addition, the mean concentration of MCs in the aerosol samples was 1.22 ng/m^3^ (ranging from 0.10 to 3.68 ng/m^3^).

**Table 1:** Concentrations of total MCs in the freshwater and aerosol samples.

Freshwater		Aerosols	
Site	MCs concentration (μg/L)	Site	MCs concentration (ng/m^3^)
FW1	15.12	AR1	0.20
FW2	5.20	AR2	0.19
FW3	366.44	AR3	3.68
FW4	70.46	AR4	0.28
FW5	5337.39	AR5-1	2.40
		AR5-2	0.10
		AR5-3	1.70

To reveal concentration of MC-producing *Microcystis* in water and aerosol samples, the levels of *Microcystis* (gene copy/100 mL of freshwater and gene copy/m^3^ of aerosol) were measured using the quantification PCR system targeting the *mcyE* gene, which is related with MC-producing genes (Table 2). The *mcyE* gene was detected in all freshwater and aerosol samples. The mean concentration of *Microcystis* (gene copies/100 mL of freshwater) in the water samples was 9.7 × 10^8^. The FW5 sample had the highest concentration of the *mcyE* gene at 2.8 × 10^9^ copies/100 mL. In the aerosol samples, the mean concentration of MC-producing *Microcystis* (gene copies/m^3^) was 1.7 × 10^1^. The highest concentration of MCs was detected from the AR1 sample (9.4 × 10^1^ gene copies m^3^).

**Table 2:** Concentrations of MC-producing *Microcystis* in the freshwater and aerosol samples.

Freshwater		Aerosols	
Site	*mcy*E concentration (copies/100 mL)	Site	*mcy*E concentration (copies/m^3^)
FW1	4.0 × 10^7^	AR1	9.4 × 10^1^
FW2	1.8 × 10^8^	AR2	1.4 × 10^0^
FW3	1.0 × 10^9^	AR3	4.2 × 10^0^
FW4	7.8 × 10^8^	AR4	1.7 × 10^0^
FW5	2.8 × 10^9^	AR5-1	7.7 × 10^0^
		AR5-2	1.2 × 10^0^
		AR5-3	5.8 × 10^0^

### 3.2. Microbial compositions of water and aerosol samples during algal bloom

**Figure 1:**
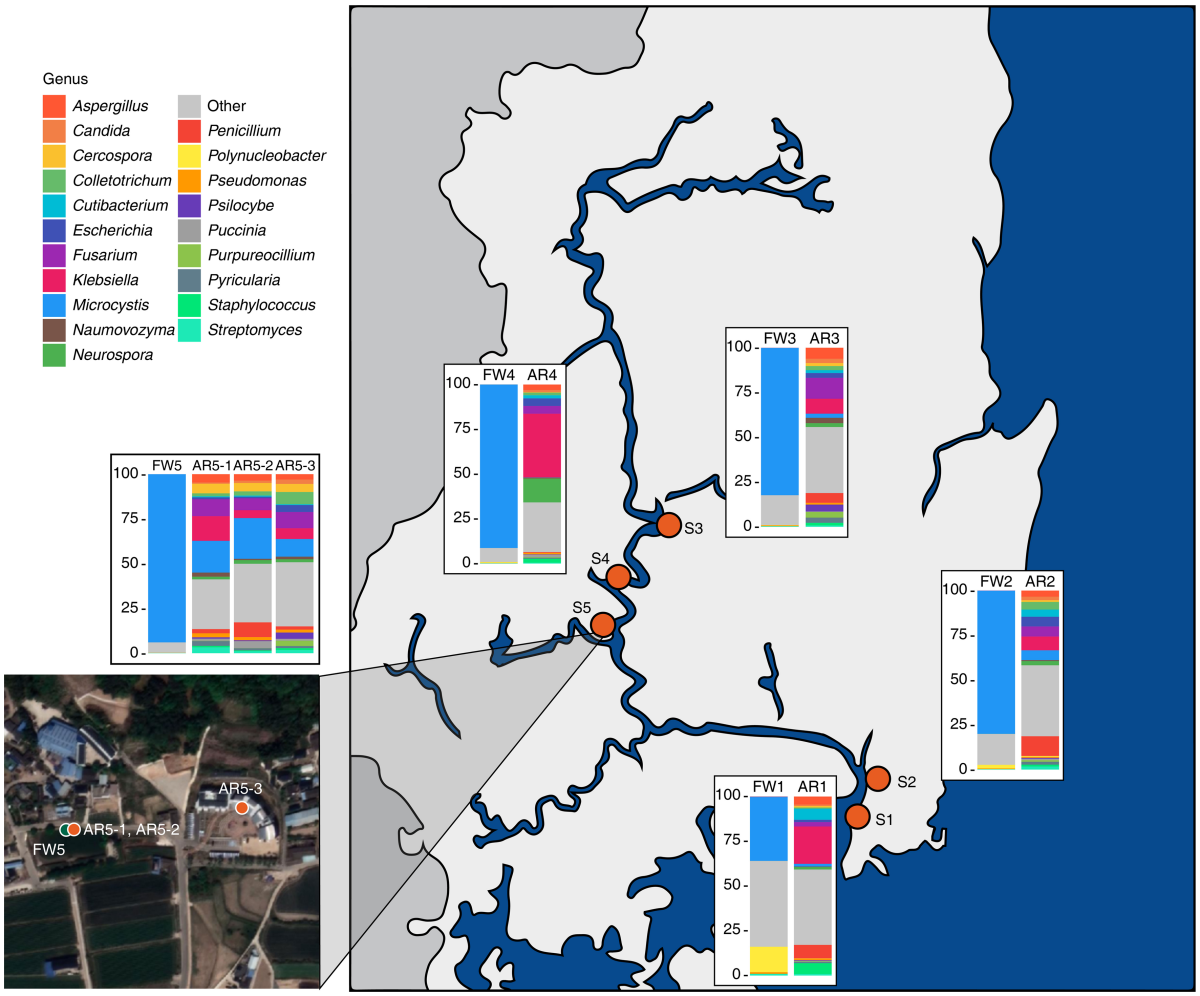
Sampling sites and the microbial composition at the genus level in the collected samples. Bar plots depicting the taxonomic composition at the genus level for both freshwater and aerosol samples across different sites.

The freshwater microbiome was dominated by *Microcystis,* but in the aerosol microbiome, various organisms, such as eukaryotes and viruses, were detected (Figure 1; Supplementary Figure S1). The freshwater microbiome was composed of 99.79% bacteria, which was composed of 76.65% *Microcystis*, followed by 0.31% *Pseudanabaena*, 0.26% *Flavobacterium*, 0.22% *Synechococcus*, and 0.19% *Planktothrix* (Figure 1; Supplementary Table S1). The aerosol microbiome was composed of 56.33% bacteria, 41.88% eukaryotes, and 1.29% viruses (Supplementary Figure S1). Especially, *Klebsiella* (13.8%) and *Microcystis* (8.54%) were dominant in the aerosol microbiome, as were fungi such as *Fusarium* (7.04%) and *Aspergillus* (4.07%) (Figure 1; Supplementary Table S1).

### 3.3. Microbial diversity and core microbiome of the freshwater and aerosols

**Figure 2:**
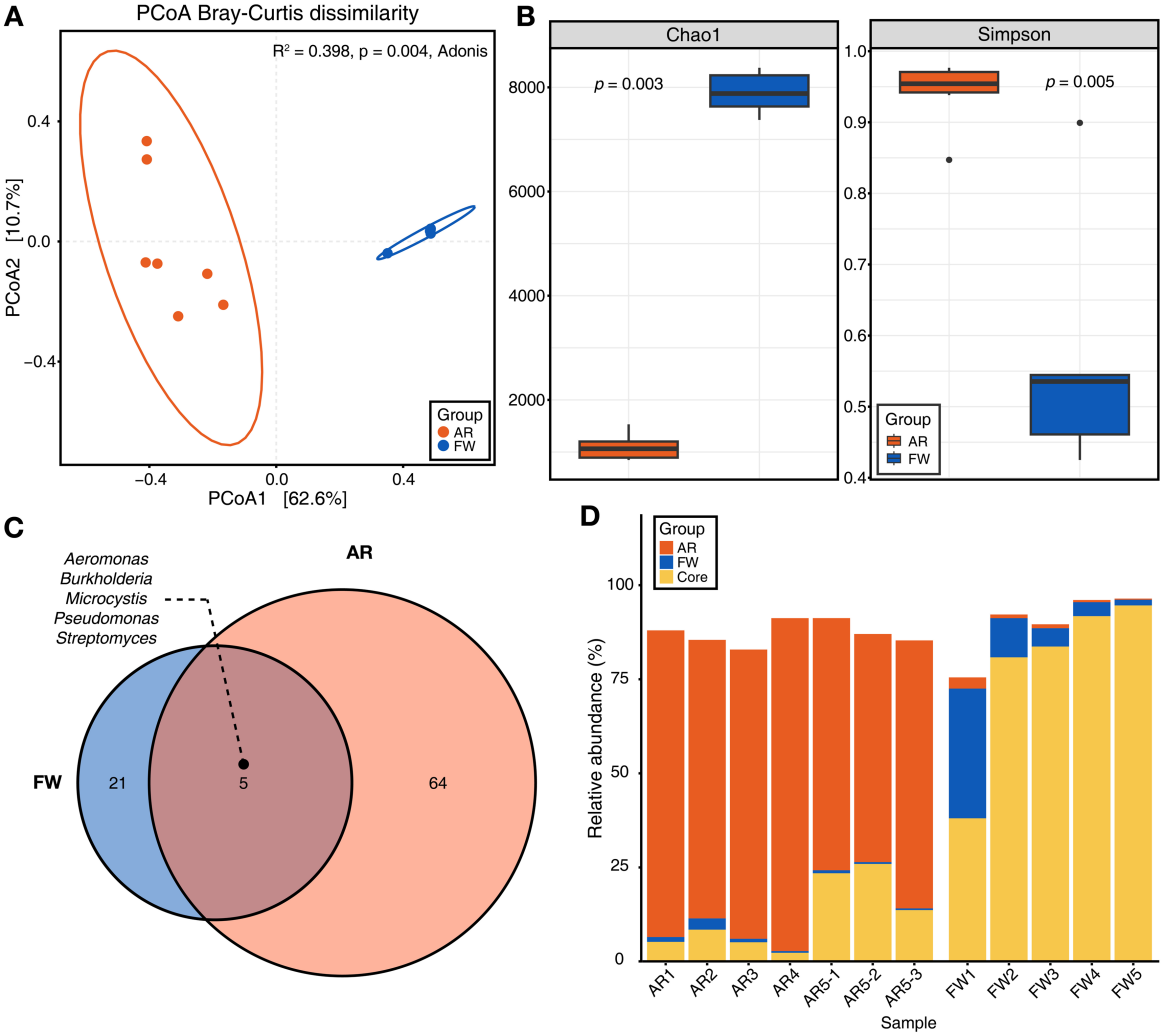
Microbial diversity and core microbiome of aerosol and freshwater. **(A)** Beta-diversity of microbiome through principal coordinates analysis (PCoA) based on Bray–Curtis dissimilarity (*p* = 0.004). **(B)** Chao1 and Simpson indices for alpha-diversity was visualized using bar plot (Wilcoxon rank sum test, *p* = 0.003 for Chao1, *p* = 0.005 for Simpson). **(C)** The Venn diagram illustrated the genera commonly found in both aerosol and freshwater samples (Detection = 0.1, Prevalence = 0.5). **(D)** Stacked bar chart displaying the relative abundance of aerosol, freshwater, and core microbiome constituents for each sample.

PCoA based on Bray–Curtis dissimilarity showed that microbial communities differed significantly between the freshwater and aerosols (Figure 2A). The aerosol microbiome had a greater variety of microbes and a higher level of evenness (Simpson’s index) than the freshwater microbiome (Figure 2B). Furthermore, the core microbiome of the each group was explored. The core microbiome refers to a set of microbial taxa that are consistently found across multiple individuals or groups, representing a stable and shared component of the overall microbiome (Neu et al., 2021). Total of five core microbiome genera, including *Aeromonas*, *Microcystis*, *Burkholderia*, *Pseudomonas*, and *Streptomyces*, were identified that were common to each groups (Figure 2C). The stacked bar chart showed the proportion of core microbiomes within each group (orange and blue), with the core microbiome shared between freshwater and aerosol samples shown in yellow. The freshwater samples are dominated by water-aerosol core microbiome (yellow), whereas the aerosol samples were predominantly composed of aerosol-unique core microbiome (orange) (Figure 2D). Biosynthetic genes of microcystin.

### 3.4. Identifying MC biosynthetic gene cluster (BGC) from freshwater and aerosols

**Figure 3:**
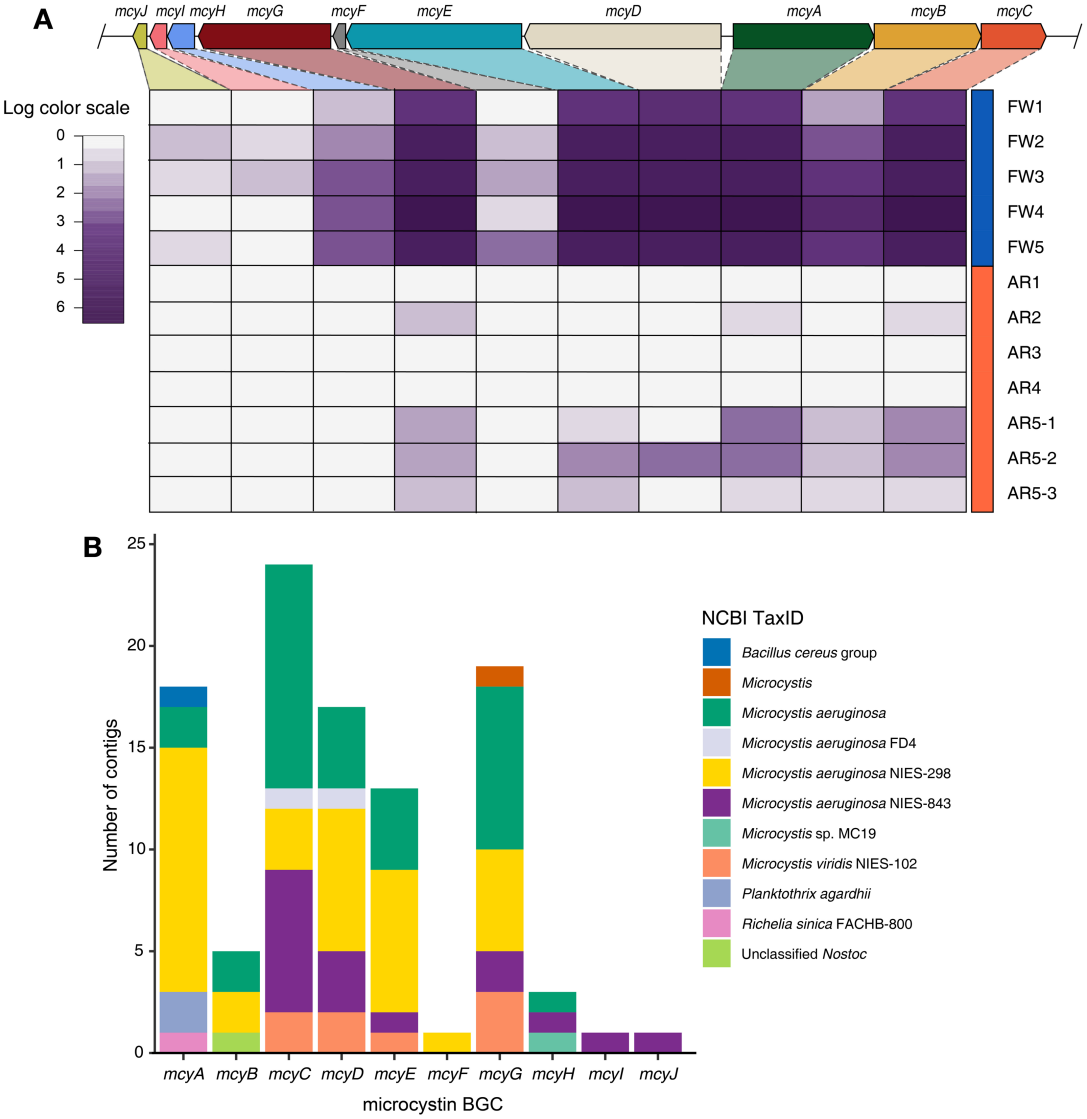
Identification of microcystin BGC and taxonomy composition of contigs. **(A)** Heatmap displaying the log-scaled abundance of the *mcy* genes in each sample. **(B)** Stacked bar plot showing the taxonomy information of contigs encoding for each *mcy* genes.

The microcystin BGC, produced by *Microcystis,* was identified in high abundance in both freshwater and aerosol samples (Figure 3A). The biosynthetic genes of microcystin (*mcyA, mcyB, mcyC, mcyD, mcyE, and mcyG*) were found in all freshwater samples, whereas these biosynthetic genes of microcystin were detected in the AR5-1, AR5-2, and AR5-3 samples (Figure 3A). Furthermore, the taxonomy of contigs annotated with each gene was examined and it is found that some contigs were classified as *Planktothrix* and *Nostoc*; however, 95.1% of the contigs were classified as *Microcystis* (Figure 3B).

### 3.5. Functional profiling of ARGs and virulence factors

**Figure 4:**
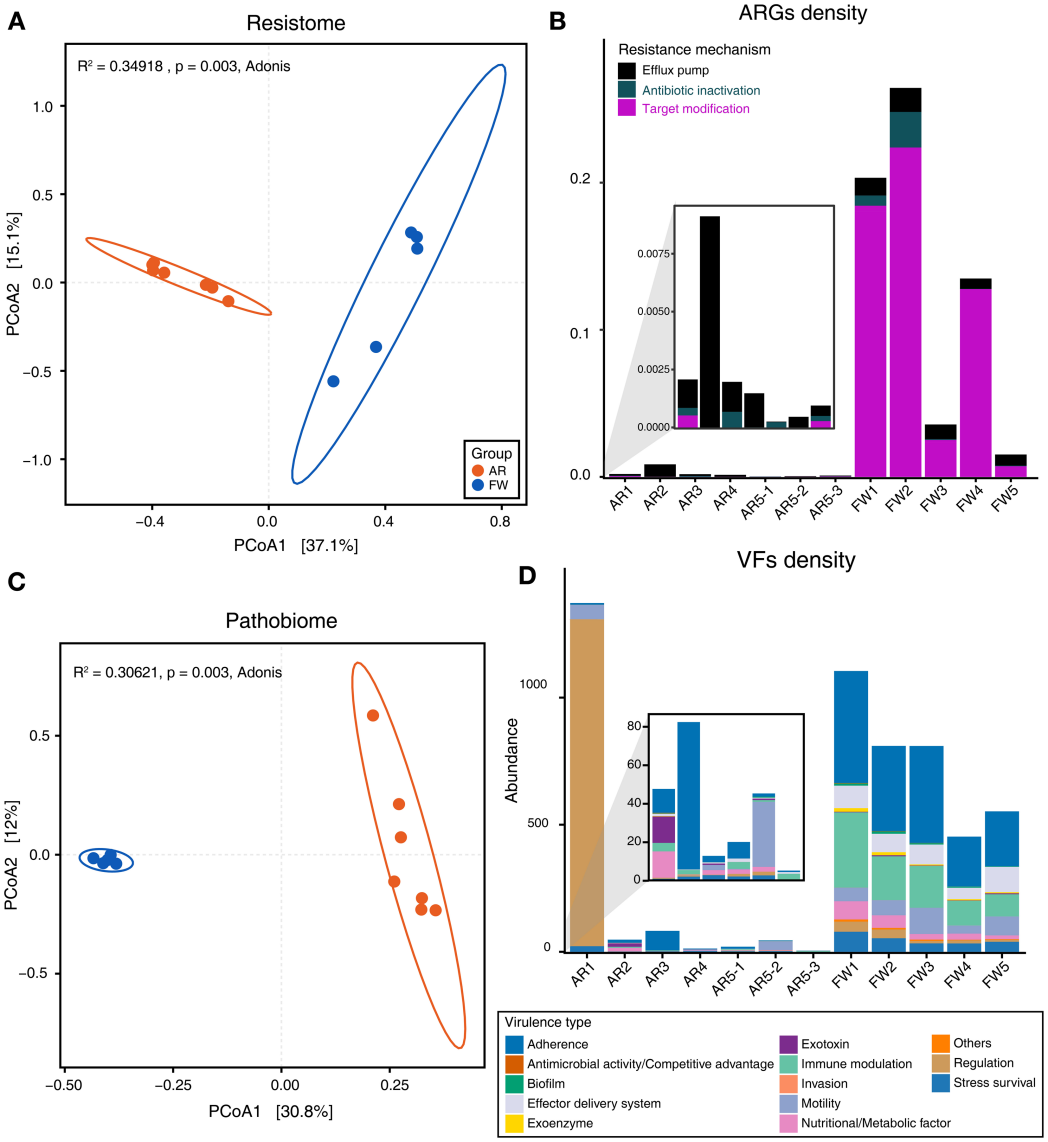
Analysis of functional diversity and density of resistome and pathobiome genes identified in AR and FW samples. **(A)** Beta-diversity of ARGs through PCoA based on Bray–Curtis dissimilarity (*p* = 0.003). **(B)** Stacked bar plot representing the density of ARGs and the proportion of each ARG type in the samples. **(C)** Beta-diversity of VFs through PCoA based on Bray–Curtis dissimilarity (*p* = 0.003). **(D)** Stacked bar plot representing the density of VFs and the proportion of each virulence type in the samples.

**Figure 5:**
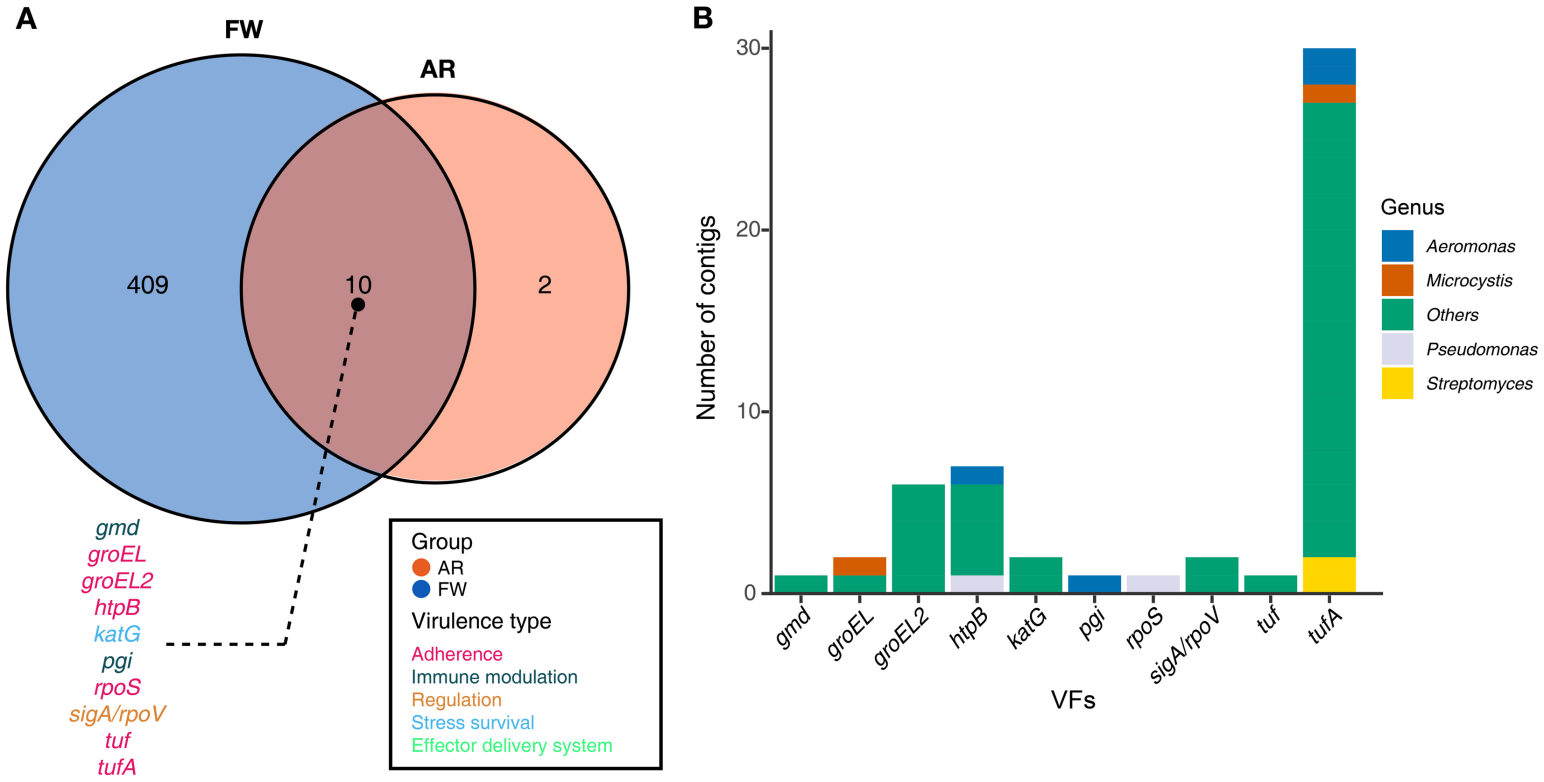
Core VFs in both groups and the taxonomic information of the contigs encoding genes. **(A)** The Venn diagram illustrated the number of core VFs found in both aerosol and freshwater samples (Detection = 0.001, Prevalence = 0.5). For the VFs present in the intersection of the Venn diagrams, the gene names were annotated using distinct colors corresponding to their respective virulence types. **(B)** A stacked bar plot illustrating the distribution of contigs encoding each gene, categorized according to taxonomic information.

**Figure 6:**
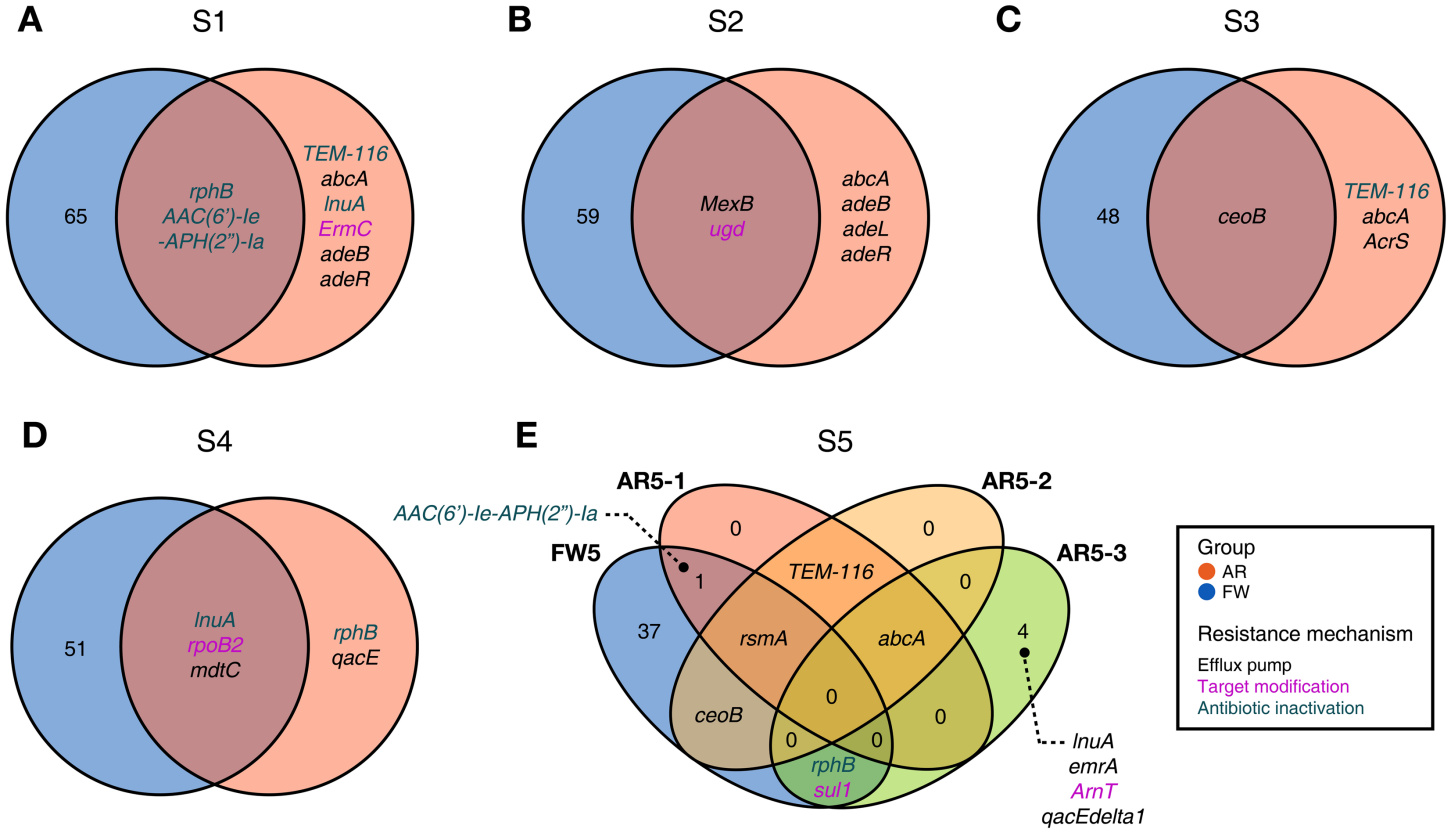
Commonly found ARGs in individual FW and AR samples at the same site. **(A-E)** The Venn diagram visualizing common ARGs in each site, colors indicating the resistance mechanism.

Functional diversities were also significantly different between freshwater and aerosol samples in both the resistome and pathobiome (Figures 4A,C). ARGs were also found in the aerosol samples, although at lower concentrations than in the freshwater samples (Figure 4B). In the freshwater samples, ARGs associated with target modification mechanisms constituted the highest percentage, whereas in the aerosol samples, ARGs with efflux pump mechanisms were more prevalent (Figure 4B). Regarding virulence factors (VFs), although they were significantly more abundant in freshwater, both groups were enriched in genes belonging to the adherence type (Figure 4D). Interestingly, the abundance of a specific gene, named *PhoQ*, was notably high in AR2 samples. Upon examining genes with a high proportion of each virulence type, we observed that the *tufA* gene had the highest abundance in the Adherence type, followed by *gmd* in the Immune Modulation type, *katB* in the Stress Survival type, and *hemB* in the Nutritional/Metabolic Factor type. (Supplementary Table 3). To identify commonly found VFs in freshwater and aerosol samples, we defined core genes as detected to 50% in each group sample. A total of ten core VFs were identified in both freshwater and aerosol samples (Figure 5A). Additionally, we analyzed the taxonomic origins of contigs harboring core VF genes and found that contigs encoding *groEL, htpB, pgi, rpoS, and tufA* genes were part of the core microbiome associated with *Aeromonas*, *Microcystis*, *Pseudomonas*, and *Streptomyces* taxa (Figure 5B). For the core ARGs, we compared the freshwater and aerosol samples at each site individually, each site had their unique core ARGs including *aac(6′)-Ie-aph(2″)-Ia, rphB, MexB, ugd, ceoB, lnuA, rpoB2, mdtC, rsmA,* and *sul1* genes (Figures 6A-E). Notably, among the core ARGs, it was confirmed that *ugd, rsmA, rpoB2, and ceoB* were presented in contigs classified as part of the core microbiome, with the exception of *Microcystis* (Supplementary Table 2).

## 4. Discussion

Due to eutrophication and increased water temperature, the increasing frequency and intensity of HABs in freshwater environments are becoming a global problem (O’Neil et al., 2012). Particularly, in recent years, there has been growing interest in the toxic factors present in aerosols emitted from freshwater bodies. For instance, Wood and Dietrich (2011) detected aerosolized MCs concentrations of up to 1.8 pg./m^3^ in two lakes in New Zealand, and Backer et al. (2010) detected an average of 0.052 ng/m^3^ of MCs in the air of bloom-affected lakes in California. Numerous studies have extensively explored the composition of microbial communities in aerosols, with a particular emphasis on harmful algae (Madikizela et al., 2018; Olson et al., 2020; Harb et al., 2021; Plaas et al., 2022). Especially, Plaas et al. (2022) identified aerosolized *Dolichospermum*, *Microcystis,* and *Aphanizomenon*. However, limited research has been conducted on the function of microbial communities that are related to pathogenicity factors (Olson et al., 2020). This study investigated the microbial communities in freshwater environments and nearby aerosol samples using metagenomic sequencing, to explore the composition and function of micro-organisms. We aimed to assess the potential hazards of aerosols generated from HAB-affected freshwater bodies with identifying profiles of pathogenic and virulence factors in microbial communities.

Our results demonstrated the taxonomic composition of the freshwater samples: 30–90% of the *Microcystis* genus and MCs were discovered at five sites in the Nakdong River basin. The *Microcystis* genus represents one of the key cyanobacteria and is known for its carcinogenic properties, hepatotoxicity, and reproductive toxicity, among other effects (Svirčev et al., 2010; Lone et al., 2015). Conversely, in the aerosol samples, *Microcystis* was not detected in large quantities across all samples but was found to be present in high proportions at specific sites (AR5-1 ~ 3). A previous study indicated that microbial diversity of aerosols varies according to climate; this difference is likely influenced by the climate (water temperature, humidity, and wind direction) at the time of sampling (Lone et al., 2015).

The microbial community diversity is markedly distinct between freshwater and aerosol samples, with the freshwater group exhibiting greater bacterial richness but significantly less evenness compared to the aerosol group. This disparity is attributable to the dominance of *Microcystis* in the freshwater group during bloom seasons. However, in the aerosol group, various micro-organisms, including bacteria, fungi, and viruses, are relatively evenly distributed. Collectively, these results indicate that the aerosol environment, which can change rapidly in response to climatic and ambient conditions, is only marginally influenced by the adjacent water microbial community. This observation aligns with the higher proportion of the aerosol-core microbiome than the aerosol-freshwater core microbiome in the taxonomic composition of the aerosol samples. Interestingly, AR5-1 ~ 3 samples exhibited a high proportion of *Microcystis*, which was consistent in neighboring freshwater samples. Among the regions identified in this study, FW5 had the highest abundance of *Microcystis*.

We examined specific genes related with MC biosynthesis (*mcy* gene) produced by toxin-producing cyanobacteria. The core genes *mcyA-E* and *mcyG*, which are responsible for the synthesis, assembly, and modification of MCs (Tillett et al., 2000), were present in high abundance in the freshwater samples. We also observed the *mcy* genes in the aerosol samples. The abundance of *mcy* genes was consistently higher in the AR 5–1, 5–2, and 5–3 samples with high *Microcystis* abundance than in other samples (e.g., AR 1–4). Furthermore, most of the contigs encoding MC BGC are classified as *Microcystis*, which suggests that *Microcystis* found in aerosols may indeed have a toxin-producing function and raises the possibility of transporting harmful substances via aerosols.

Furthermore, the genera *Aeromonas*, *Burkholderia*, *Pseudomonas*, and *Streptomyces* were identified as common components of the core microbiome. These Gram-negative bacteria form biofilms and are highly adapted and widespread in various environments due to their remarkable metabolic versatility (Seshadri et al., 2006; Rojo, 2010; Kaltenpoth and Flórez, 2020; Kumar et al., 2023). Consequently, they have the potential to colonize multiple environments when dispersed through aerosols. Additionally, the presence of these four genera among the contigs encoding ARGs implies the potential existence of ARG carriers within the core microbiome. Indeed, *Aeromonas* and *Burkholderia* are already well established as ARG carriers in river (Esiobu et al., 2002; Piotrowska and Popowska, 2014). Although no direct evidence exists to attribute the presence of these core microbiomes in aerosol samples to freshwater sources, microbes found in freshwater samples are also commonly detected in aerosol samples, and these microbes have pathogenic characteristics that can cause dissemination of harmful agents through aerosols.

In addition, VFs and ARGs were analyzed to determine the distribution of potential harmful factors in the aerosol microbiome. Both types of genes exhibited significant differences between the aerosol and freshwater groups, with freshwater samples displaying higher gene abundance than aerosol samples. However, ARGs and VFs were also detected in the aerosol samples, albeit at low concentrations. Previous studies have demonstrated that aerosol samples can be dispersed over considerable distances, indicating a potential risk of harmful agents in aerosols spreading to humans (Gorbunov, 2020). Benson et al. (2005) suggested that trace amounts of MCs could potentially induce adverse effects when MCs are introduced into the human respiratory tract via aerosols. Moreover, the acute toxicity of MCs may be more potent when exposure occurs via inhalation rather than ingestion (Creasia, 1990). A major contributor to the proliferation and outbreak of ARGs is the misuse of antibiotics (Shallcross and Davies, 2014). Although antibiotics are extensively employed in human, livestock, and agricultural settings (Madikizela et al., 2018; Chang et al., 2019; Tian et al., 2021), to our knowledge, they are not applied to or transported through the air. Nonetheless, the detection of ARGs in aerosols can be attributed to antibiotic-resistant bacteria carrying ARGs. Indeed, prior studies have reported the detection of ARGs in aerosols near livestock barns, where ARGs are frequently found (Li et al., 2019). Given the detection of ARGs in aerosols around freshwater environments, ARGs present in aerosols in areas with minimal human activity are likely to have been transferred from freshwater environments, which are known as ARG reservoirs.

Furthermore, a total of ten VFs were identified as common in both AR and FW samples, among which the *groEL*, *groEL2*, *htpB*, *rpoS*, *tuf*, and *tufA* genes are of the adherence type. If bacteria carrying these functional genes become aerosolized, they may play a crucial role in the subsequent attachment of aerosolized bacteria to host cells or surfaces, a critical step in establishing infections (Hennequin et al., 2001; Srivastava et al., 2008). Moreover, in accordance with previous findings, the presence of VFs in core microbiome taxa suggests that harmful, functional microbes indeed exist in aerosols.

## 5. Conclusion

In this preliminary study, we employed metagenomic sequencing to explore the microbial communities within aerosols and their associated MC-producing genes, which have not yet been thoroughly investigated. Aerosols have been inadequately studied due to challenges in sample collection and their susceptibility to the surrounding environment. However, recent outbreaks of COVID-19 and pneumonia infections (*Mycobacterium tuberculosis*), both prevalent nosocomial infections, are transmitted via aerosols (Li et al., 2020; Borges et al., 2021). Furthermore, the major limitation of this study is a lack of collecting field blanks, which is for ensuring the quality of aerosol samples, concurrently during the day. However, in this study, collection of blank samples was conducted on a clean bench under controlled laboratory conditions, potentially influencing the results. For a future study, it is necessary to consider measuring the blanks in the field under the same conditions to guarantee the accuracy of the data.

Consequently, monitoring harmful microorganisms and their genes in aerosols is essential. This study underscores the importance of aerosol metagenomics by identifying detrimental microorganisms and genes in aerosols from environments with minimal human interaction. Moreover, because of the limited data on contributing factors and the small sample size in this study, we could not establish a direct link between freshwater environments and aerosols. Therefore, future research is needed to confirm the transfer of harmful microorganisms from freshwater environments to aerosols.

## Data availability statement

The datasets presented in this study are deposited in NCBI SRA online repositories under BioProject accession number PRJNA949880 (https://www.ncbi.nlm.nih.gov/bioproject/PRJNA949880).

## Author contributions

J-HS and SL designed study, supervised the project, and edited the manuscript. JK and SH collected data and samples. JK, SH, GL, and M-JK analysed data, performed statistical analyses, and wrote the draft. GL and M-JK conducted sequencing. All authors contributed to the article and approved the submitted version.

## Funding

This research was supported by a project to train professional personnel in biological materials by the Ministry of Environment and the Korea Basic Science Institute (National Research Facilities and Equipment Center) grant funded by the Ministry of Education (2021R1A6C101A416). This work was also supported by the National Research Foundation of Korea (NRF) grant funded by the Korea government (MSIT) (No. 2022R1C1C1008799). This work was supported by the Pukyong National University Research Fund in 2021(CD20210981).

## Conflict of interest

The authors declare that the research was conducted in the absence of any commercial or financial relationships that could be construed as a potential conflict of interest.

## Publisher’s note

All claims expressed in this article are solely those of the authors and do not necessarily represent those of their affiliated organizations, or those of the publisher, the editors and the reviewers. Any product that may be evaluated in this article, or claim that may be made by its manufacturer, is not guaranteed or endorsed by the publisher.

## References

[ref1] Alcock B. P. Raphenya A. R. Lau T. T. Y. Tsang K. K. Bouchard M. Edalatmand A.. (2019). CARD 2020: antibiotic resistome surveillance with the comprehensive antibiotic resistance database. Nucleic Acids Res. 48, D517–D525. doi: 10.1093/nar/gkz935, PMID: 31665441 PMC7145624

[ref2] Backer L. C. Mcneel S. V. Barber T. Kirkpatrick B. Williams C. Irvin M.. (2010). Recreational exposure to microcystins during algal blooms in two California lakes. Toxicon 55, 909–921. doi: 10.1016/j.toxicon.2009.07.006 19615396

[ref200] Bass D. Stentiford G. D. Wang H. C. Koskella B. Tyler C. R. (2019). The pathobiome in animal and plant diseases. Trends Ecol. Evol 43(11), 996–1008. doi: 10.1016/j.tree.2019.07.012, PMID: 31522755 PMC7479508

[ref3] Benson J. M. Hutt J. A. Rein K. Boggs S. E. Barr E. B. Fleming L. E. (2005). The toxicity of microcystin LR in mice following 7 days of inhalation exposure. Toxicon 45, 691–698. doi: 10.1016/j.toxicon.2005.01.004, PMID: 15804518 PMC2551753

[ref4] Bhattacharya R. Sugendran K. Dangi R. Rao P. (1997). Toxicity evaluation of freshwater cyanobacterium *Microcystis aeruginosa* PCC 7806: II nephrotoxicity in rats. Biomed. Environ. Sci. 10, 93–101. PMID: 9099431

[ref5] Bolger A. M. Lohse M. Usadel B. (2014). Trimmomatic: a flexible trimmer for Illumina sequence data. Bioinformatics 30, 2114–2120. doi: 10.1093/bioinformatics/btu170, PMID: 24695404 PMC4103590

[ref6] Borges J. T. Nakada L. Y. K. Maniero M. G. Guimarães J. R. (2021). SARS-CoV-2: a systematic review of indoor air sampling for virus detection. Environ. Sci. Pollut. Res. 28, 40460–40473. doi: 10.1007/s11356-021-13001-w, PMID: 33630259 PMC7905194

[ref7] Buchfink B. Xie C. Huson D. H. (2015). Fast and sensitive protein alignment using DIAMOND. Nat. Methods 12, 59–60. doi: 10.1038/nmeth.3176, PMID: 25402007

[ref8] Bushnell B. (2014). "BBMap: a fast, accurate, splice-aware aligner". In *9th annual genomics of Energy & Environment Meeting*. 17-20 march, University of California. 2014.

[ref9] Chang Y. Chusri S. Sangthong R. McNeil E. Hu J. Du W.. (2019). Clinical pattern of antibiotic overuse and misuse in primary healthcare hospitals in the southwest of China. PLoS One 14:e0214779. doi: 10.1371/journal.pone.0214779, PMID: 31242185 PMC6594576

[ref11] Choi A. -R. Oh H. -M. Lee J. -A. (2002). Ecological study on the toxic *Microcystis* in the lower Nakdong River. Algae 17, 171–185. doi: 10.4490/ALGAE.2002.17.3.171

[ref12] Creasia D. (1990). Acute inhalation toxicity of microcystin-LR with mice. Toxicon 28:605.

[ref13] Dawson R. M. (1998). The toxicology of microcystins. Toxicon 36, 953–962. doi: 10.1016/S0041-0101(97)00102-5 9690788

[ref14] Esiobu N. Armenta L. Ike J. (2002). Antibiotic resistance in soil and water environments. Int. J. Environ. Health Res. 12, 133–144. doi: 10.1080/09603120220129292 12396530

[ref16] Gonçalves A. L. Rodrigues C. M. Pires J. C. M. Simões M. (2016). The effect of increasing CO_2_ concentrations on its capture, biomass production and wastewater bioremediation by microalgae and cyanobacteria. Algal Res. 14, 127–136. doi: 10.1016/j.algal.2016.01.008

[ref17] Gorbunov B. (2020). Aerosol particles laden with viruses that cause COVID-19 travel over 30m distance. *Preprints.org* [preprint] (2020). Available at: doi: 10.20944/preprints202004.0546.v1

[ref18] Hachicha R. Elleuch F. Ben Hlima H. Dubessay P. De Baynast H. Delattre C.. (2022). Biomolecules from microalgae and Cyanobacteria: applications and market survey. Appl. SciApplied Sciences. 12:1924. doi: 10.3390/app12041924

[ref700] Harb C. Pan J. DeVilbiss S. Badgley B. Marr L. C. Schmale D. G. III Foroutan H. (2021). Increasing freshwater salinity impacts aerosolized bacteria. Environ. Sci. Technol 55(9), 5731–5741. doi: 10.1021/acs.est.0c08558, PMID: 33819033

[ref19] Hennequin C. Porcheray F. Waligora-Dupriet A. -J. Collignon A. Barc M. -C. Bourlioux P.. (2001). GroEL (Hsp60) of *Clostridium difficile* is involved in cell adherence. Microbiology 147, 87–96. doi: 10.1099/00221287-147-1-87, PMID: 11160803

[ref20] Hur M. Lee I. Tak B. -M. Lee H. J. Yu J. J. Cheon S. U.. (2013). Temporal shifts in cyanobacterial communities at different sites on the Nakdong River in Korea. Water Res. 47, 6973–6982. doi: 10.1016/j.watres.2013.09.058, PMID: 24169512

[ref21] Ito E. Kondo F. Terao K. Harada K. -I. (1997). Neoplastic nodular formation in mouse liver induced by repeated intraperitoneal injections of microcystin-LR. Toxicon 35, 1453–1457. doi: 10.1016/S0041-0101(97)00026-3, PMID: 9403968

[ref22] Kaltenpoth M. Flórez L. V. (2020). Versatile and dynamic symbioses between insects and Burkholderia bacteria. Annu. Rev. Entomol. 65, 145–170. doi: 10.1146/annurev-ento-011019-025025, PMID: 31594411

[ref23] Kim H. G. Hong S. Chon T. -S. Joo G. -J. (2021). Spatial patterning of chlorophyll a and water-quality measurements for determining environmental thresholds for local eutrophication in the Nakdong River basin. Environ. Pollut. 268:115701. doi: 10.1016/j.envpol.2020.115701, PMID: 33045591

[ref24] Kim S. Chung S. Park H. Cho Y. Lee H. (2019). Analysis of environmental factors associated with Cyanobacterial dominance after river weir installation. Water. 11:1163. doi: 10.3390/w11061163

[ref25] Kumar M. Tiwari P. Zeyad M. T. Ansari W. A. Kumar S. C. Chakdar H.. (2023). Genetic diversity and antifungal activities of the genera *Streptomyces* and *Nocardiopsis* inhabiting agricultural fields of Tamil Nadu India. J. King Saud Univ. Sci. 35:102619. doi: 10.1016/j.jksus.2023.102619

[ref27] Labohá P. Sychrová E. Brózman O. Sovadinová I. Bláhová L. Prokeš R.. (2023). Cyanobacteria, cyanotoxins and lipopolysaccharides in aerosols from inland freshwater bodies and their effects on human bronchial cells. Environ. Toxicol. Pharmacol. 98:104073. doi: 10.1016/j.etap.2023.104073, PMID: 36738853

[ref28] Lad A. Breidenbach J. D. Su R. C. Murray J. Kuang R. Mascarenhas A.. (2022). As we drink and breathe: adverse health effects of microcystins and other harmful algal bloom toxins in the liver, gut, lungs and beyond. Life. 12:418. doi: 10.3390/life12030418, PMID: 35330169 PMC8950847

[ref29] Lee H. -J. Park H. -K. Cheon S. -U. (2018). Effects of weir construction on phytoplankton assemblages and water quality in a large river system. Int. J. Environ. Res. Public Health 15:2348. doi: 10.3390/ijerph15112348, PMID: 30356004 PMC6265701

[ref30] Lee S. Kim J. Lee J. (2021). Colonization of toxic cyanobacteria on the surface and inside of leafy green: a hidden source of cyanotoxin production and exposure. Food Microbiol. 94:103655. doi: 10.1016/j.fm.2020.103655, PMID: 33279080

[ref31] Li D. Liu C. -M. Luo R. Sadakane K. Lam T. -W. (2015). MEGAHIT: an ultra-fast single-node solution for large and complex metagenomics assembly via succinct de Bruijn graph. Bioinformatics 31, 1674–1676. doi: 10.1093/bioinformatics/btv033, PMID: 25609793

[ref32] Li J. Zhang L. Ren Z. Xing C. Qiao P. Chang B. (2020). Meteorological factors correlate with transmission of 2019-nCoV: proof of incidence of novel coronavirus pneumonia in Hubei Province, China. *Medrxiv* [Preprint] (2020). Available at. doi: 10.1101/2020.04.01.20050526

[ref33] Li W. Godzik A. (2006). Cd-hit: a fast program for clustering and comparing large sets of protein or nucleotide sequences. Bioinformatics 22, 1658–1659. doi: 10.1093/bioinformatics/btl158 16731699

[ref34] Li W. Mao F. Te S. H. He Y. Gin K. Y. H. (2021). Impacts of *Microcystis* on the dissemination of the antibiotic resistome in cyanobacterial blooms. ACS ES&T Water. 1, 1263–1273. doi: 10.1021/acsestwater.1c00006

[ref35] Li Y. Liao H. Yao H. (2019). Prevalence of antibiotic resistance genes in air-conditioning systems in hospitals, farms, and residences. Int. J. Environ. Res. Public Health 16:683. doi: 10.3390/ijerph16050683, PMID: 30813565 PMC6427721

[ref36] Liu B. Zheng D. Zhou S. Chen L. Yang J. (2022). VFDB 2022: a general classification scheme for bacterial virulence factors. Nucleic Acids Res. 50, D912–D917. doi: 10.1093/nar/gkab1107, PMID: 34850947 PMC8728188

[ref37] Lone Y. Koiri R. K. Bhide M. (2015). An overview of the toxic effect of potential human carcinogen microcystin-LR on testis. Toxicol. Rep. 2, 289–296. doi: 10.1016/j.toxrep.2015.01.008, PMID: 28962362 PMC5598424

[ref38] Lu J. Breitwieser F. P. Thielen P. Salzberg S. L. (2017). Bracken: estimating species abundance in metagenomics data. PeerJ. Comput. Sci. 3:e104. doi: 10.7717/peerj-cs.104 PMC12016282 40271438

[ref39] Lu J. Struewing I. Wymer L. Tettenhorst D. R. Shoemaker J. Allen J. (2020). Use of qPCR and RT-qPCR for monitoring variations of microcystin producers and as an early warning system to predict toxin production in an Ohio inland lake. Water Res. 170:115262. doi: 10.1016/j.watres.2019.115262, PMID: 31785564 PMC7075668

[ref40] Madikizela L. M. Ncube S. Chimuka L. (2018). Uptake of pharmaceuticals by plants grown under hydroponic conditions and natural occurring plant species: a review. Sci. Total Environ. 636, 477–486. doi: 10.1016/j.scitotenv.2018.04.297, PMID: 29709865

[ref900] Murby A. L. Haney J. F. (2016). Field and laboratory methods to monitor lake aerosols for cyanobacteria and microcystins. Aerobiologia, 32, 395–403.

[ref600] Neu A. T. Allen E. E. Roy K. (2021). Defining and quantifying the core microbiome: Challenges and prospects. Proc. Natl. Acad. Sci., 118(51), e2104429118. doi: 10.1073/pnas.2104429118 34862327 PMC8713806

[ref41] O’Neil J. M. Davis T. W. Burford M. A. Gobler C. J. (2012). The rise of harmful cyanobacteria blooms: the potential roles of eutrophication and climate change. Harmful Algae 14, 313–334. doi: 10.1016/j.hal.2011.10.027

[ref42] Olson N. E. Cooke M. E. Shi J. H. Birbeck J. A. Westrick J. A. Ault A. P. (2020). Harmful algal bloom toxins in aerosol generated from inland lake water. Environ. Sci. Technol. 54, 4769–4780. doi: 10.1021/acs.est.9b07727, PMID: 32186187 PMC11406200

[ref43] Oren A. (2011). Cyanobacterial systematics and nomenclature as featured in the international bulletin of bacteriological nomenclature and taxonomy/international journal of systematic bacteriology/international journal of systematic and evolutionary microbiology. Int. J. Syst. Evol. Microbiol. 61, 10–15. doi: 10.1099/ijs.0.018838-0, PMID: 21097637

[ref44] Orrell J. (2022). Risk analysis of aerosolized algae atmospheric transport in northwestern Ohio from the western basin of Lake Erie. [master’s thesis]. [Columbus (OH)]: The Ohio State University.

[ref45] Palinska K. A. Surosz W. (2014). Taxonomy of cyanobacteria: a contribution to consensus approach. Hydrobiologia 740, 1–11. doi: 10.1007/s10750-014-1971-9

[ref46] Park H. -K. Lee H. -J. Heo J. Yun J. -H. Kim Y. -J. Kim H. -M.. (2021). Deciphering the key factors determining spatio-temporal heterogeneity of cyanobacterial bloom dynamics in the Nakdong River with consecutive large weirs. Sci. Total Environ. 755:143079. doi: 10.1016/j.scitotenv.2020.143079, PMID: 33127129

[ref47] Piotrowska M. Popowska M. (2014). The prevalence of antibiotic resistance genes among Aeromonas species in aquatic environments. Ann. Microbiol. 64, 921–934. doi: 10.1007/s13213-014-0911-2

[ref48] Plaas H. E. Paerl R. W. Baumann K. Karl C. Popendorf K. J. Barnard M. A.. (2022). Harmful cyanobacterial aerosolization dynamics in the airshed of a eutrophic estuary. Sci. Total Environ. 852:158383. doi: 10.1016/j.scitotenv.2022.158383, PMID: 36057302

[ref50] Rogers M. M. Stanley R. K. (2023). Airborne algae: a rising public health risk. Environ. Sci. Technol, 57, 5501–5503. doi: 10.1021/acs.est.3c01158, PMID: 36996349 PMC10100814

[ref51] Rojo F. (2010). Carbon catabolite repression in *Pseudomonas*: optimizing metabolic versatility and interactions with the environment. FEMS Microbiol. Rev. 34, 658–684. doi: 10.1111/j.1574-6976.2010.00218.x, PMID: 20412307

[ref52] Ryu H. -S. Park H. -K. Lee H. -J. Shin R. -Y. Cheon S. -U. (2016). Occurrence and succession pattern of Cyanobacteria in the upper region of the Nakdong River: factors influencing Aphanizomenon bloom. J. Korean Soc. Water Environ. 32, 52–59. doi: 10.15681/KSWE.2016.32.1.52

[ref53] Schaefer A. M. Yrastorza L. Stockley N. Harvey K. Harris N. Grady R.. (2020). Exposure to microcystin among coastal residents during a cyanobacteria bloom in Florida. Harmful Algae 92:101769. doi: 10.1016/j.hal.2020.101769, PMID: 32113588

[ref54] Seshadri R. Joseph S. W. Chopra A. K. Sha J. Shaw J. Graf J.. (2006). Genome sequence of *Aeromonas hydrophila* ATCC 7966^T^: jack of all trades. J. Bacteriol. 188, 8272–8282. doi: 10.1128/JB.00621-06, PMID: 16980456 PMC1698176

[ref55] Shallcross L. J. Davies D. S. C. (2014). Antibiotic overuse: a key driver of antimicrobial resistance. Br. J. Gen. Pract. 64, 604–605. doi: 10.3399/bjgp14X682561, PMID: 25452508 PMC4240113

[ref56] Sharma N. K. Rai A. K. Singh S. Brown R. M. Jr. (2007). Airborne algae: their present status and relevance^1^. J. Phycol. 43, 615–627. doi: 10.1111/j.1529-8817.2007.00373.x

[ref57] Sipari H. Rantala-Ylinen A. Jokela J. Oksanen I. Sivonen K. (2010). Development of a Chip assay and quantitative PCR for detecting microcystin Synthetase E gene expression. Appl. Environ. Microbiol. 76, 3797–3805. doi: 10.1128/AEM.00452-10, PMID: 20400558 PMC2893508

[ref58] Smith V. H. Schindler D. W. (2009). Eutrophication science: where do we go from here? Trends Ecol, 24, 201–207. doi: 10.1016/j.tree.2008.11.009, PMID: 19246117

[ref59] Srivastava S. Yadav A. Seem K. Mishra S. Chaudhary V. Nautiyal C. (2008). Effect of high temperature on *Pseudomonas putida* NBRI0987 biofilm formation and expression of stress sigma factor RpoS. Curr. Microbiol. 56, 453–457. doi: 10.1007/s00284-008-9105-0, PMID: 18219523

[ref60] Stewart I. Webb P. M. Schluter P. J. Shaw G. R. (2006). Recreational and occupational field exposure to freshwater cyanobacteria – a review of anecdotal and case reports, epidemiological studies and the challenges for epidemiologic assessment. Environ. Health 5:6. doi: 10.1186/1476-069X-5-6, PMID: 16563159 PMC1513208

[ref61] Svirčev Z. Baltić V. Gantar M. Juković M. Stojanović D. Baltić M. (2010). Molecular aspects of microcystin-induced hepatotoxicity and hepatocarcinogenesis. J. Environ. Sci. Health C 28, 39–59. doi: 10.1080/10590500903585382, PMID: 20390967

[ref500] Swe T. Miles C. O. Cerasino L. Mjelde M. Kleiven S. Ballot A. (2021). Microcystis, Raphidiopsis raciborskii and Dolichospermum smithii, toxin producing and non-toxigenic cyanobacteria in Yezin Dam, Myanmar. Limnologica 90, 125901. doi: 10.1016/j.limno.2021.125901, PMID: 20390967

[ref62] Tesson S. V. M. Skjøth C. A. Šantl-Temkiv T. Löndahl J. (2016). Airborne microalgae: insights, opportunities, and challenges. Appl. Environ. Microbiol. 82, 1978–1991. doi: 10.1128/AEM.03333-15, PMID: 26801574 PMC4807511

[ref63] Tian M. He X. Feng Y. Wang W. Chen H. Gong M.. (2021). Pollution by antibiotics and antimicrobial resistance in livestock and poultry manure in China, and countermeasures. Antibiotics. 10:539. doi: 10.3390/antibiotics10050539, PMID: 34066587 PMC8148549

[ref64] Tillett D. Dittmann E. Erhard M. Von Döhren H. Börner T. Neilan B. A. (2000). Structural organization of microcystin biosynthesis in *Microcystis aeruginosa* PCC7806: an integrated peptide–polyketide synthetase system. Chem. Biol. 7, 753–764. doi: 10.1016/S1074-5521(00)00021-1, PMID: 11033079

[ref400] Vigar M. Thuneibat M. Jacobi A. Roberts V. A. (2022). Summary report–One Health Harmful Algal Bloom System (OHHABS). Centers for Disease Control and Prevention. Available at: https://www.cdc.gov/habs/data/2020-ohhabs-data-summary.html

[ref300] Wilson N. (2022). Nutrient and sediment dynamics in two coastal plain rivers in the Bay of Plenty (Master dissertation, The University of Waikato). available at: https://researchcommons.waikato.ac.nz/handle/10289/15218

[ref66] Wood D. E. Lu J. Langmead B. (2019). Improved metagenomic analysis with kraken 2. Genome Biol. 20, 257–213. doi: 10.1186/s13059-019-1891-0, PMID: 31779668 PMC6883579

[ref67] Wood S. A. Dietrich D. R. (2011). Quantitative assessment of aerosolized cyanobacterial toxins at two New Zealand lakes. J. Environ. Monitor. 13, 1617–1624. doi: 10.1039/C1EM10102A, PMID: 21491044

[ref68] Zhang Q. Zhang Z. Lu T. Peijnenburg W. J. G. M. Gillings M. Yang X.. (2020). Cyanobacterial blooms contribute to the diversity of antibiotic-resistance genes in aquatic ecosystems. Commun. Biol. 3:737. doi: 10.1038/s42003-020-01468-1, PMID: 33277584 PMC7718256

[ref69] Zhao C. S. Shao N. F. Yang S. T. Ren H. Ge Y. R. Feng P.. (2019). Predicting cyanobacteria bloom occurrence in lakes and reservoirs before blooms occur. Sci. Total Environ. 670, 837–848. doi: 10.1016/j.scitotenv.2019.03.161, PMID: 30921717

