# Supplementary material for: Microbial communities in aerosol generated from cyanobacterial bloom-affected freshwater bodies: an exploratory study in Nakdong River, South Korea
Source: Front Microbiol. 2023 Jul 13;14:1203317. doi: 10.3389/fmicb.2023.1203317 (PMC10374321; doi:10.3389/fmicb.2023.1203317)
Supplement: Supplementary file 1 [file Data_Sheet_1.zip › Supplementary Material contents.pdf]

Supplementary Material

Microbial Communities in Aerosol Generated from Cyanobacterial Bloom-affected Freshwater Bodies: an Exploratory Study in Nakdong River, South Korea

Jinnam Kim[1,†], GyuDae Lee[2, †], Soyoung Han[1], Min-Ji Kim[2], Jae-Ho Shin[2,3,*], Seungjun Lee[1,*]

[1] Major of Food Science & Nutrition, Division of Food Science, College of Fisheries Science, Pukyong National University, Busan, Republic of Korea

[2] Department of Applied Biosciences, Kyungpook National University, Daegu, Republic of Korea

[3] NGS Core Facility, Kyungpook National University, Daegu, Republic of Korea

[†] These authors share first authorship

*** Correspondence:** Seungjun Leepaul, 5280@pknu.ac.kr

Jae-Ho Shin, jhshin@knu.ac.kr

1 Supplementary Table

Supplementary Table 1. Relative abundance of microbiome at the genus level

Supplementary Table 2. Taxonomic information of contigs encoding common antibiotic resistance genes (ARGs)

Supplementary Table 3. Information about the type of each virulence factor genes (RPKM per samples)

2 Supplementary Figures

Supplementary Figure 1. Pie chart depicting the composition of microorganisms at the kingdom level for both freshwater **(A)** and aerosol **(B)** groups
