# Supplementary figures and images for: Microbial communities in aerosol generated from cyanobacterial bloom-affected freshwater bodies: an exploratory study in Nakdong River, South Korea
Source: Front Microbiol. 2023 Jul 13;14:1203317. doi: 10.3389/fmicb.2023.1203317 (PMC10374321; doi:10.3389/fmicb.2023.1203317)

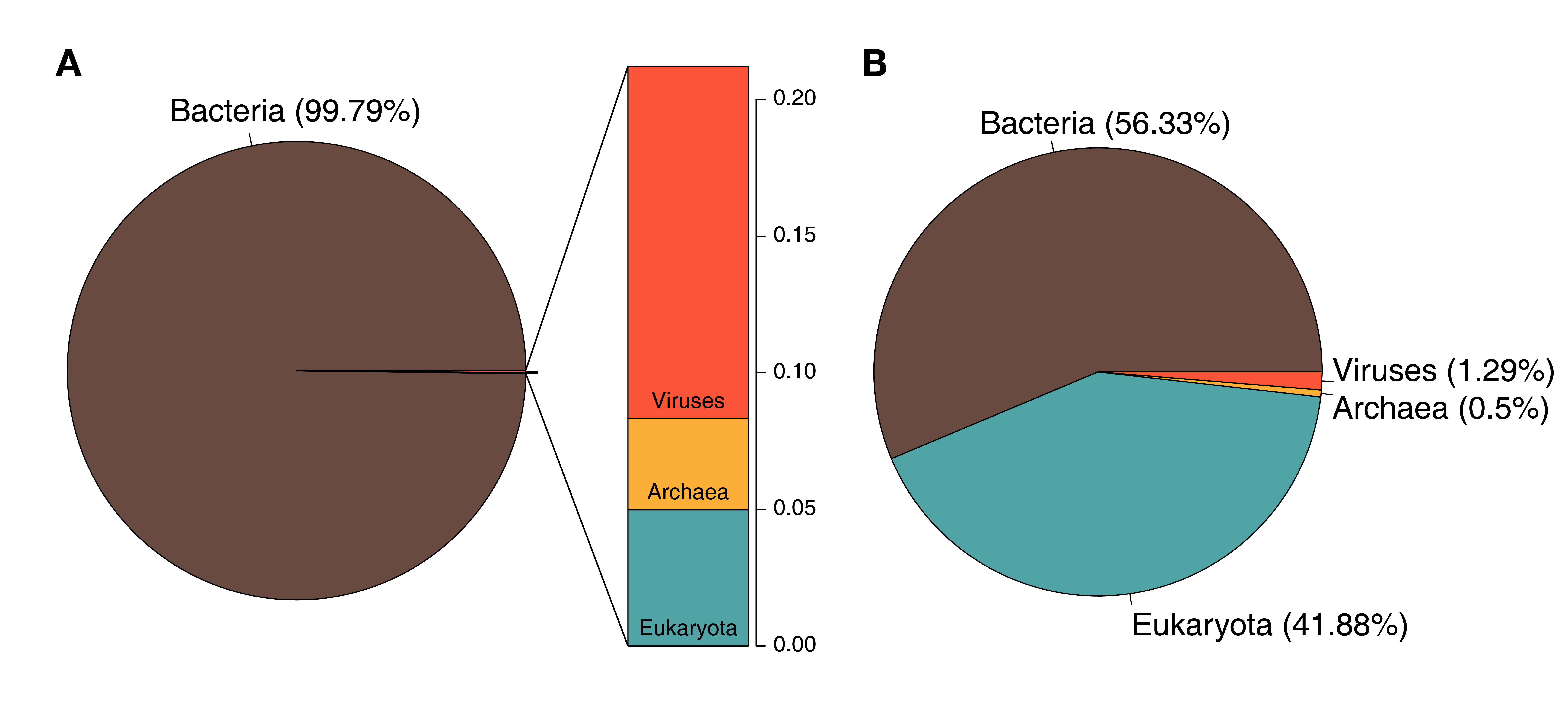

Supplement: Supplementary file 1 [file Data_Sheet_1.zip › Supplementary Figure S1.JPEG]
